# Phase I Trial of Combination Therapy With Avelumab and Cabozantinib in Patients With Newly Diagnosed Metastatic Clear Cell Renal Cell Carcinoma

**DOI:** 10.1093/oncolo/oyad019

**Published:** 2023-03-23

**Authors:** Haoran Li, Kamal Kant Sahu, James Brundage, Mallory Benson, Umang Swami, Kenneth M Boucher, Sumati Gupta, Josiah Hawks, Deepika Sirohi, Neeraj Agarwal, Benjamin L Maughan

**Affiliations:** Division of Medical Oncology, Department of Internal Medicine, Huntsman Cancer Institute, University of Utah, Salt Lake City, UT, USA; Division of Medical Oncology, Department of Internal Medicine, Huntsman Cancer Institute, University of Utah, Salt Lake City, UT, USA; Division of Medical Oncology, Department of Internal Medicine, Huntsman Cancer Institute, University of Utah, Salt Lake City, UT, USA; Division of Medical Oncology, Department of Internal Medicine, Huntsman Cancer Institute, University of Utah, Salt Lake City, UT, USA; Division of Medical Oncology, Department of Internal Medicine, Huntsman Cancer Institute, University of Utah, Salt Lake City, UT, USA; Department of Internal Medicine, Huntsman Cancer Institute, University of Utah, Salt Lake City, UT, USA; Division of Medical Oncology, Department of Internal Medicine, Huntsman Cancer Institute, University of Utah, Salt Lake City, UT, USA; Division of Medical Oncology, Department of Internal Medicine, Huntsman Cancer Institute, University of Utah, Salt Lake City, UT, USA; Department of Pathology, ARUP Institute for Clinical and Experimental Pathology, Huntsman Cancer Institute, University of Utah, Salt Lake City, UT, USA; Division of Medical Oncology, Department of Internal Medicine, Huntsman Cancer Institute, University of Utah, Salt Lake City, UT, USA; Division of Medical Oncology, Department of Internal Medicine, Huntsman Cancer Institute, University of Utah, Salt Lake City, UT, USA

**Keywords:** renal cell carcinoma, avelumab, cabozantinib, PD L-1, tyrosine kinase inhibitor

## Abstract

**Background:**

Combination immunotherapy is now considered the standard first-line therapy for patients with metastatic clear cell renal cell carcinoma (mccRCC) after multiple clinical trials demonstrated improved overall survival compared with single-agent tyrosine kinase inhibitors. Cabozantinib modulates critical components of the immune system, such as decreasing regulatory T cells and increasing T-effector cell populations, and is approved for the treatment of mRCC. Avelumab is a human IgG1 monoclonal antibody that binds to programmed death-ligand 1 protein and inhibits the interaction with PD-1. This phase I trial assessed the safety and clinical activity of avelumab and cabozantinib combination therapy in mccRCC.

**Methods:**

This study was a phase I, 3+3 dose escalation clinical trial. The primary endpoint was the safety and identification of the recommended phase II dose (RP2D). Secondary endpoints included objective response rate (ORR) and radiographic progression-free survival (rPFS). There were 3 dose cohorts: cabozantinib 20, 40, and 60 mg/day, each combined with avelumab (10 mg/kg intravenously every 2 weeks). An additional 3 patients were included in the final dose cohort as a confirmation of the RP2D. No dose modifications were allowed for avelumab, but dose delays were permitted. Both dose reductions and holds were allowed for cabozantinib. Response Evaluation Criteria in Solid Tumors (RECIST), version 1.1, was used to determine ORR, and treatment beyond progression was allowed.

**Results:**

Twelve patients with newly diagnosed mccRCC were enrolled from July 2018 until March 2020. Three patients were enrolled in the 20 and 40 mg cohorts each, and 6 were enrolled in the 60 mg cohort. The International Metastatic RCC Database Consortium (IMDC) risk categories for these patients were: 4 patients (favorable risk), 6 patients (intermediate risk), and 2 patients (poor risk). No dose-limiting toxicities (DLTs) were observed in any cohort. Six patients developed serious adverse events related to study treatment after the DLT window period. Immune-related adverse events (iRAEs) were reported in 11 patients; fatigue and diarrhea were the most common (each with *n* = 4, 33.3%), followed by maculopapular rash and hand-foot syndrome (each with *n* = 3, 25%). Dose reductions were required in 5 of 6 patients in the cabozantinib 60 mg cohort after the DLT period. One patient discontinued avelumab due to irAE (nephritis), while none discontinued cabozantinib due to toxicity. The ORR was 50%, with one complete response (CR) and 5 partial responses (PR). The disease control rate (CR + PR + stable disease) was noted in 92% of the patients. Radiological PFS survival rate at 6 and 12 months was reported in 67.7% and 33.5% of patients, respectively.

**Conclusion:**

Combination therapy with avelumab and cabozantinib is safe and showed preliminary clinical activity in mccRCC. Even though the DLT was not met in any of the 3 cohorts, the recommended RP2D dose for the combination is cabozantinib 40 mg/day due to a high incidence of grade 2 toxicity for cabozantinib 60 mg/day after the DLT period. (ClinicalTrials.gov Identifier: NCT03200587)

## Teaser

Combination immunotherapy is now considered standard first-line therapy for patients with metastatic clear cell renal cell carcinoma. This phase I trial assessed the safety and clinical activity of avelumab and cabozantinib combination therapy.

**Lessons Learned**

- The recommended dose of cabozantinib in combination with avelumab is 40 mg daily.
- Avelumab and cabozantinib achieved disease control rate and objective response rate of 92% and 50%, respectively, in untreated metastatic renal cell carcinoma.
- The combination therapy of avelumab and cabozantinib is well tolerated with manageable side effects.

## Discussion

The therapeutic landscape for patients with metastatic clear cell renal cell carcinoma (mccRCC) has rapidly expanded.^1^ Nivolumab, a programmed cell death protein-1 (PD-1) inhibitor, was the first immune checkpoint inhibitor (ICI) U.S. Food and Drug Administration (FDA) approved for mRCC based on the CheckMate 025 trial.^2^ In 2016, cabozantinib, a tyrosine kinase inhibitor (TKI), also received FDA approval for treatment in the first-line setting based on results from the CABOSUN trial.^3^ Since then, various combination regimens of ICIs and antiangiogenic agents have been studied and approved for mccRCC.^4-6^

Cabozantinib is an inhibitor of multiple tyrosine kinases, including vascular endothelial growth factor receptor 1-3 (VEGF), hepatocyte growth factor receptor (MET), AXL, RET, KIT, FLT3, ROS1, MER, TYRO3, TRKB, and TIE-2.^7^ In addition, cabozantinib has immunomodulatory characteristics that neutralize cancer-induced immunosuppression.^8^ These characteristics possibly explain the additive clinical activity of cabozantinib with nivolumab observed in the Checkmate 9ER trial.^6^

Avelumab is a fully human immunoglobulin G1 (IgG1) monoclonal antibody against programmed death-ligand 1 (PD-L1), which is present on the cancer cell surface. Avelumab is different from the other drugs in the same class for its unique characteristic of antitumor activity via natural killer (NK) cell-mediated antibody-dependent cell-mediated cytotoxicity (ADCC).^9^ Based on the results of JAVELIN Renal 101 study, the combination of avelumab plus axitinib is approved for use as first-line agent for mRCC.

**Figure 1.**
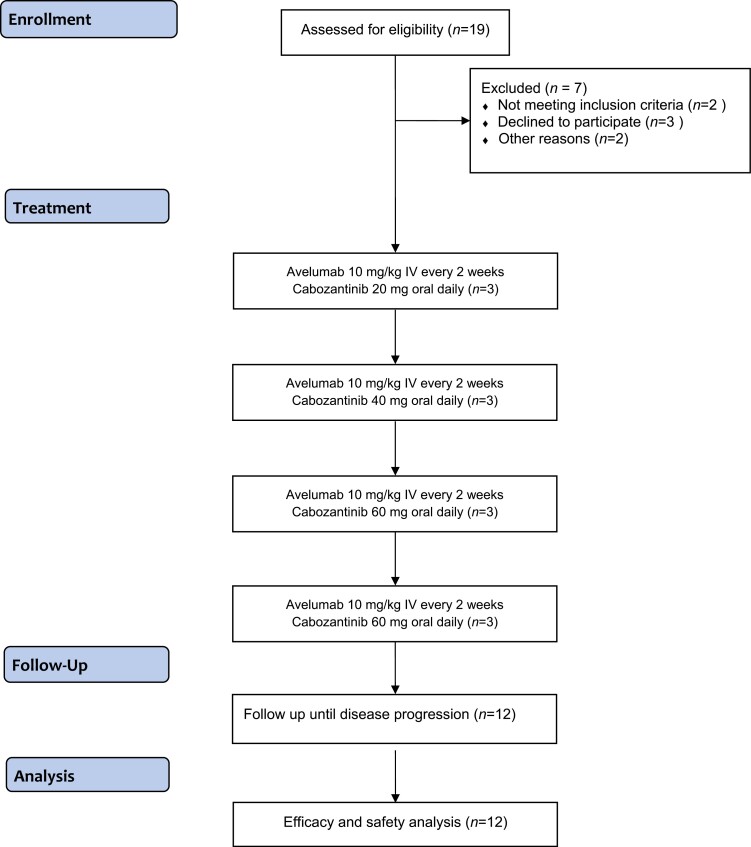
CONSORT flow diagram showing the enrollment of subjects, and their allocation to treatment.

This study evaluated the safety and efficacy of a novel combination of avelumab and cabozantinib in untreated mccRCC (Fig. 1). There were no dose-limiting toxicities. The safety data is consistent with other clinical trials of VEGF/TKI combinations in mRCC. Due to the high incidence of grade 2 toxicities for cabozantinib at 60 mg/day, the RP2D dose for cabozantinib is 40 mg/day. We also observed an ORR of 50% and a DCR of 92%, suggesting significant clinical activity of this combination. This is the first study to provide safety and efficacy data for this novel combination, which is being tested in a large phase III trial as the maintenance therapy in metastatic urothelial carcinoma.^10^

**Table:** 

Trial Information	
Disease	Metastatic clear cell renal cell carcinoma
Stage of disease/treatment	Metastatic disease/ combination therapy with avelumab and cabozantinib
Prior therapy	None
Type of study	Phase I, 3 + 3
Primary endpoints	To determine the safety of the combined use of avelumab plus cabozantinib and to identify the recommended phase II dose of cabozantinib in patients with mccRCC.
Secondary endpoints	Investigator-assessed confirmed objective response rate, disease control rate, and radiological progression-free survival.
Investigator’s analysis	Active but results were overtaken by other developments

## Additional Details of Endpoints or Study Design

### Patients and Methods

#### Patients

Eligible patients were adults with histologically proven and previously untreated mRCC with a clear cell component. Patients had measurable disease according to Response Evaluation Criteria in Solid Tumors (RECIST), version 1.1. Patients with any International Metastatic Renal-Cell Carcinoma Database Consortium (IMDC) prognostic risk score were included. An Eastern Cooperative Oncology Group (ECOG) performance status score of 0-2 as assessed by the investigator and radiographic evidence of metastatic disease/stage IV (American Joint Committee on Cancer, AJCC) were included. Patients with active autoimmune disease, active use of immunosuppressive medications, prior exposure to ICIs or cabozantinib in the adjuvant setting, or symptomatic venous thrombosis were excluded from the study. Incidentally detected asymptomatic deep venous thrombosis (DVT) or pulmonary embolism (PE) on routine scans were allowed if stable and treated with therapeutic anticoagulation for at least 2 weeks before the first dose of therapy.

#### Trial Design and Treatment

This was a phase I trial of combination therapy with avelumab and cabozantinib in patients with newly diagnosed mccRCC. Dose-escalation was performed using a standard 3 + 3 design with cabozantinib oral doses of 20, 40, and 60 mg/day plus avelumab 10 mg/kg every 2 weeks in each of the 3 cohorts (Fig. 1). A pre-planned dose expansion cohort of 3 additional patients was included in the final dose cohort as a confirmation of the recommended phase II dose (RP2D). Dose reductions were allowed for cabozantinib but not for avelumab. Dose delays were permitted for both medications to manage adverse events (AEs). AEs and laboratory abnormalities were classified and graded according to the National Cancer Institute (NCI) Common Terminology Criteria for Adverse Events (CTCAE) version 4.0. Dose-limiting toxicity (DLT) was defined as any grade ≥ 3 AE occurring during the first 3 weeks of treatment and considered related to either of the investigational drugs as determined by the investigator. The maximum tolerated dose (MTD) was defined as the highest dose level at which ≤ 1 of 6 evaluable patients experienced a DLT. Patients were treated until disease progression or unacceptable toxicity or until other protocol-specified criteria for withdrawal were met. Treatment was discontinued for any grade 4 AE, except for single laboratory values out of the normal range that was deemed unrelated to study treatment, without clinical correlation, and that resolved in ≤ 7 days with medical management. Patients could receive treatment beyond progression if felt to be clinically benefiting per the treating investigator.

Safety assessments included documentation of AEs, physical examination, clinical laboratory tests (hematology labs, hepatic panels, and serum chemistries), and documentation of concurrent medications. A serious adverse event (SAE) was defined as any untoward event that resulted in death, life-threatening, required inpatient hospitalization, prolongation of existing hospitalization, resulted in persistent or significant disability/incapacity, a congenital anomaly/birth defect, or was otherwise considered medically necessary. Immune-related AEs (irAEs) were identified using a pre-specified list of Medical Dictionary for Regulatory Activities (MedDRA) terms, followed by a comprehensive medical review. Infusion-related reactions (IRRs) were analyzed using both a prespecified list of MedDRA preferred terms (reactions occurring post-infusion on the same day or following day) and related signs and symptoms (based on specified MedDRA terms) that occurred on the day of infusion and resolved in ≤ 2 days.

#### Outcomes

The primary objective of this phase I dose-escalation trial was to determine the safety of the combined use of avelumab plus cabozantinib and to identify the RP2D in patients with mccRCC.

The secondary objective was to define the preliminary clinical activity of the combination drug regimen. Secondary endpoints included: investigator-assessed confirmed objective response rate (ORR defined as the proportion of patients who achieve CR or PR using RECIST v1.1), disease control rate (DCR defined as the proportion of patients who achieve complete response [CR], partial response [PR], or stable disease [SD]), and radiological PFS. Radiographic tumor assessments were performed at baseline and repeated every 6 weeks. The post hoc analysis included reporting the duration of treatment, defined as the time from initiation of therapy until the start of next-line line therapy or death.

#### Statistical Analysis

Patient demographics and baseline characteristics were described using frequencies and percentages (%) for categorical variables and medians and interquartile ranges for continuous variables. Safety and clinical activity (ORR, DCR, and radiological PFS) were analyzed in all patients. Estimates of the percentage of patients with objective response and the exact 2-sided 95% CI were computed according to the exact Clopper-Pearson method. Response duration and PFS were estimated using the Kaplan-Meier method.

**Table:** 

Drug Information		
	Drug 1	Drug 2
Generic/working name	Cabozantinib	Avelumab
Company name	Exelixis	Merck, and Pfizer
Drug type	Small-molecule inhibitor	Monoclonal antibody
Drug class	Tyrosine kinase inhibitor	Programmed death-ligand 1 inhibitor
Dose	20, 40, and 60 mg/day	10 mg/kg every 2 weeks
Unit	mg/day	mg/kg
Route	Oral	Intravenous (i.v.)
Schedule of Administration	Every day	Every 2 weeks

**Table:** 

Dose Escalation Table			
**Cohort**	**Dose of drug** **Cabozantinib**	**Dose of drug** **Avelumab**	**Number enrolled**
Cohort 1	20 mg/kg/day (oral)	10 mg/kg (i.v.) every 2 weeks	3
Cohort 2	40 mg/kg/day (oral)	10 mg/kg (i.v.) every 2 weeks	3
Cohort 3	60 mg/kg/day (oral)	10 mg/kg ( i.v.) every 2 weeks	6

**Table:** 

Patient Characteristics	
Number of patients, male	7
Number of patients, female	5
Stage	IV
Age: median (range)	66.5 (47-81) years
Number of prior systemic therapies	0
Performance status: ECOG	0: 6
	1: 6
	2: 0
	3: 0
	4: 0
Cancer types or histologic subtypes	Clear cell subtype: 12 (100%)
Notes	In the dose escalation cohort, a total of 9 patients received avelumab 10 mg/kg every 2 weeks in combination with cabozantinib at doses of 20 mg (*n* = 3), 40 mg (*n* = 3), or 60 mg (*n* = 3) daily. An additional 3 patients were included in the 60 mg/day dose expansion cohort. Patients were enrolled at a single site (Huntsman Cancer Institute at the University of Utah) from August 2018 until March 2020. The baseline demographics and clinical characteristics are summarized in Table 1 . The median age of patients in our cohort was 66.5 years, with 7 (58.3%) patients being male. IMDC risk stratification of the patients was as follows: favorable in 4 (33.3%), intermediate in 6 (50%), and poor risk in 2 (16.7%) patients. All patients had 2 or more organs involved with metastatic cancer. None of them had received any systemic therapy before enrollment in this study.

**Table:** 

Primary Assessment Method	
Title	Dose-limiting toxicity and maximum tolerated dose
Number of patients screened	19
Number of patients enrolled	12
Number of patients evaluable for toxicity	12
Number of patients evaluated for efficacy	12
Evaluation method	RECIST 1.1

## Outcome Notes

### Safety

#### AEs (Regardless of Treatment Attribution)

The incidence of adverse events (AEs) is summarized in Table 2. Combining all the 3 cohorts, diarrhea (91.6%), thromboembolic events (91.6%), and palmar-plantar erythrodysesthesia syndrome (75%) were the 3 most common adverse effects of any grade noted during the study period. Similarly, combining all the 3 cohorts, the 3 most common grade 3 or more adverse events noted were thromboembolic events (41.6%), diarrhea (25%), and hypertension (25%).

**Table 1.** Baseline characteristics of patients.

Characteristics	Number of patients (%)
Median age [range] in years	66.5 [47-81]
Male gender	7 (58.3)
Caucasian	12 (100)
Clear cell subtype	12 (100)
Stage IV	12 (100)
Number of organs with metastases	
1	0
≥2	12 (100)
Bone metastasis	4 (33.3)
Liver metastasis	1 (8.3)
Brain metastasis	1 (8.3)
Lactate dehydrogenase at baseline, median (range), U/L	210.5 (98-1672)
History of venous thrombosis	1 (8.3)
History of tumor thrombus	3 (25)
History of hypertension	10 (83.3)
History of stroke	0
History of myocardial infarction	0
Prior systemic therapies	0
Prior nephrectomy	5 (42)
IMDC prognostic risk	
Favorable	4 (33.3)
Intermediate	6 (50)
Poor	2 (16.7)
From diagnosis to systemic therapy < 1 year	8 (66.7)
Karnofsky performance status <80%	0
Anemia	6 (50)
Hypercalcemia	1 (8.3)
Neutrophilia	1 (8.3)
Thrombocytosis	1 (8.3)

**Table 2.** All incidences of adverse events (regardless of attribution).

	Cabozantinib 20 mg plus avelumab (*n* = 3)		Cabozantinib 40 mg plus avelumab (*n* = 3)		Cabozantinib 60 mg plus avelumab (*n* = 6)		Total (n-=12)	
Adverse events, *n* (%)	Any grade	Grades 3-5	Any grade	Grades 3-5	Any grade	Grades 3-5	Any grade	Grades^€^ 3-5
Diarrhea	3 (100)	0	2 (66.7)	1 (33.3)	6 (100)	2 (33.3)	11 (91.6)	3 (25)
Thromboembolic events^¥^	1 (33.3)	1 (33.3)	4 (100)	2 (66.7)	6 (100)	2 (33.3)	11 (91.6)	5 (41.6)
Palmar-plantar erythrodysesthesia syndrome	1 (33.3)	0	2 (66.7)	0	6 (100)	1 (16.7)	9 (75)	1 (8.3)
Dysgeusia	1 (33.3)	0	2 (66.7)	0	4 (66.7)	0	7 (58.3)	0
Mucositis oral	1 (33.3)	0	1 (33.3)	0	4 (66.7)	1 (16.7)	6 (50)	1 (8.3)
Anorexia	1 (33.3)	0	1 (33.3)	0	3 (50)	1 (16.7)	5 (41.6)	1 (8.3)
Hypothyroidism	1 (33.3)	0	1 (33.3)	0	3 (50)	0	5 (41.6)	0
Fatigue			2 (66.7)	0	3 (50)	1 (16.7)	5 (41.6)	1 (8.3)
Hypokalemia					3 (50)	1 (16.7)	3 (25)	1 (8.3)
Vomiting					3 (50)	0	3 (25)	0
Weight loss	1 (33.3)	0	2 (66.7)	0	2 (33.3)	0	5 (41.6)	0
Skin ulceration	1 (33.3)	0	1 (33.3)	0	2 (33.3)	0	4 (33.3)	0
ALT increased	1 (33.3)	0			2 (33.3)	1 (16.7)	3 (25)	1 (8.3)
AST increased	1 (33.3)	0			2 (33.3)	1 (16.7)	3 (25)	1 (8.3)
Hoarseness	1 (33.3)	0			2 (33.3)	0	3 (25)	0
Hypertension	1 (33.3)	1 (33.3)			2 (33.3)	2 (33.3)	3 (25)	3 (25)
Infections			1 (33.3)	0	2 (33.3)	1 (16.7)	3 (25)	1 (8.3)
Nausea			1 (33.3)	0	2 (33.3)	0	3 (25)	0
Acute kidney injury					2 (33.3)	1 (16.7)	2 (16.7)	1 (8.3)
Headache					2 (33.3)	0 (0)	2 (16.7)	0
Hyponatremia					2 (33.3)	2 (33.3)	2 (16.7)	2 (16.7)
Pain	2 (66.7)	0	2 (66.7)	0	1 (16.7)	0	5 (41.6)	0
Rash maculopapular	1 (33.3)	0	3 (100)	1 (33.3)	1 (16.7)	0	5 (41.6)	1 (8.3)
Cough	2 (66.7)	0	1 (33.3)	0	1 (16.7)	0	4 (33.3)	0
Dry skin	2 (66.7)	0			1 (16.7)	0	3 (25)	0
Flatulence	2 (66.7)	0			1 (16.7)	0	3 (25)	0
Edema limbs	1 (33.3)	0	1 (33.3)	0	1 (16.7)	0	3 (25)	0
Myalgia	1 (33.3)	0	1 (33.3)	0	1 (16.7)	0	3 (25)	0
Fall		0	2 (66.7)	1 (33.3)	1 (16.7)	0	3 (25)	1 (8.3)
Abdominal pain	1 (33.3)	0			1 (16.7)	0	2 (16.7)	0
Alopecia	1 (33.3)	0			1 (16.7)	0	2 (16.7)	0
Dry mouth	1 (33.3)	0			1 (16.7)	0	2 (16.7)	0
Insomnia	1 (33.3)	0			1 (16.7)	0	2 (16.7)	0
Oral dysesthesia	1 (33.3)	0			1 (16.7)	0	2 (16.7)	0
Postnasal drip	1 (33.3)	0			1 (16.7)	0	2 (16.7)	0
Pruritus	1 (33.3)	0			1 (16.7)	0	2 (16.7)	0
Skin and subcutaneous tissue disorders	1 (33.3)	0			1 (16.7)	0	2 (16.7)	0
Bloating			1 (33.3)	0	1 (16.7)	0	2 (16.7)	0
Colitis			1 (33.3)	1 (33.3)	1 (16.7)	0	2 (16.7)	1 (8.3)
Epistaxis			1 (33.3)	0	1 (16.7)	0	2 (16.7)	0
Gastroesophageal reflux disease			1 (33.3)	0	1 (16.7)	0	2 (16.7)	0
Hematoma			1 (33.3)	0	1 (16.7)	0	2 (16.7)	0
Skin infection			1 (33.3)	0	1 (16.7)	1 (16.7)	2 (16.7)	1 (8.3)
Agitation					1 (16.7)	0	1 (8.3)	0
Allergic rhinitis					1 (16.7)	0	1 (8.3)	0
Blurred vision					1 (16.7)	0	1 (8.3)	0
Chills					1 (16.7)	0	1 (8.3)	0
Conduction disorder					1 (16.7)	1 (16.7)	1 (8.3)	1 (8.3)
Depression					1 (16.7)	0	1 (8.3)	0
Dyspepsia					1 (16.7)	0	1 (8.3)	0
Fever					1 (16.7)	0	1 (8.3)	0
Flu-like symptoms					1 (16.7)	0	1 (8.3)	0
Hypercalcemia					1 (16.7)	1 (16.7)	1 (8.3)	1 (8.3)
Hypocalcemia					1 (16.7)	1 (16.7)	1 (8.3)	1 (8.3)
Hypotension					1 (16.7)	0	1 (8.3)	0
Lip infection					1 (16.7)	0	1 (8.3)	0
Lung infection					1 (16.7)	1 (16.7)	1 (8.3)	1 (8.3)
Nail discoloration					1 (16.7)	0	1 (8.3)	0
Paresthesia					1 (16.7)	0	1 (8.3)	0
Pain of skin					1 (16.7)	0	1 (8.3)	0
Paresthesia					1 (16.7)	0	1 (8.3)	0
Sepsis					1 (16.7)	1 (16.7)	1 (8.3)	1 (8.3)
Skin hyperpigmentation					1 (16.7)	0	1 (8.3)	0
Tooth infection					1 (16.7)	0	1 (8.3)	0
Urinary urgency					1 (16.7)	0	1 (8.3)	0
Constipation	2 (66.7)	0	2 (66.7)	0 (0)			4 (33.3)	0
Back pain	2 (66.7)	0					2 (16.7)	0
Hematuria	2 (66.7)	0					2 (16.7)	0
Anemia	1 (33.3)	1 (33.3)	1 (33.3)	1 (33.3)			2 (16.7)	2 (16.7)
Flank pain	1 (33.3)	0	1 (33.3)	0			2 (16.7)	0
Dizziness			2 (66.7)	0			2 (16.7)	0
APTT prolonged	1 (33.3)	1 (33.3)					1 (8.3)	1 (8.3)
Anxiety	1 (33.3)	0					1 (8.3)	0
Arthralgia	1 (33.3)	0					1 (8.3)	0
Bone pain	1 (33.3)	0					1 (8.3)	0
Creatinine increased	1 (33.3)	0					1 (8.3)	0
Gastrointestinal disorders	1 (33.3)	0					1 (8.3)	0
Plantar Fascitis	1 (33.3)	0					1 (8.3)	0
Nasal congestion	1 (33.3)	0					1 (8.3)	0
Periodontal disease	1 (33.3)	0					1 (8.3)	0
Platelet count decreased	1 (33.3)	0					1 (8.3)	0
Skin hypopigmentation	1 (33.3)	0					1 (8.3)	0
Sore throat	1 (33.3)	0					1 (8.3)	0
Stomach pain	1 (33.3)	0					1 (8.3)	0
Surgical and medical procedures	1 (33.3)	0					1 (8.3)	0
Syncope	1 (33.3)	1 (33.3)					1 (8.3)	1 (8.3)
Urinary tract obstruction	1 (33.3)	1 (33.3)					1 (8.3)	1 (8.3)
Urinary tract pain	1 (33.3)	0					1 (8.3)	0
Wound infection	1 (33.3)	0					1 (8.3)	0
Bruising			1 (33.3)	0			1 (8.3)	0
Dyspnea			1 (33.3)	0			1 (8.3)	0
Fracture			1 (33.3)	0			1 (8.3)	0
Hypoglycemia			1 (33.3)	0			1 (8.3)	0
Hypokalemia			1 (33.3)	0			1 (8.3)	0
Papulopustular rash			1 (33.3)	0			1 (8.3)	0
Face rash			1 (33.3)	0			1 (8.3)	0
Superficial thrombophlebitis			1 (33.3)	0			1 (8.3)	0
Upper respiratory infection			1 (33.3)	0			1 (8.3)	0
Urinary tract infection			1 (33.3)	0			1 (8.3)	0

¥= there was a total of 11 events that affected 8 patients (3 patients had 2 events each), €= There were no grade 5 events in any patient.

#### AEs Attributed to Treatment

##### AEs Attributed to Cabozantinib

All patients (*n* = 12) developed one or more AEs that was attributed to cabozantinib, but most were grades 1-2 (Table 3).

**Table 3.** Incidence of treatment-related adverse events attributed to cabozantinib.

	Cabozantinib 20 mg plus avelumab (*n* = 3)		Cabozantinib 40 mg plus avelumab (*n* = 3)		Cabozantinib 60 mg plus avelumab (*n* = 6)		Total (*n* = 12)	
Adverse events, *n* (%)	Any grade	Grades 3-5	Any grade	Grades 3-5	Any grade	Grades 3-5	Any grade	Grades^€^ 3-5
Diarrhea	3 (100)		1 (33.3)	1 (33.3)	6 (100)	1 (16.7)	10 (83.3)	2 (16.7)
Palmar-plantar erythrodysesthesia syndrome	1 (33.3)		2 (66.7)		5 (83.3)	1 (16.7)	8 (66.7)	1 (8.3)
Thromboembolic events	1 (33.3)	1 (33.3)	2 (66.7)	2 (66.7)	5 (83.3)	2 (33.3)	8 (58.3)	5 (25)
Dysgeusia	1 (33.3)		2 (66.7)		4 (66.7)		7 (58.3)	
Mucositis oral	1 (33.3)		1 (33.3)		4 (66.7)	1 (16.7)	6 (50)	1 (8.3)
Anorexia	1 (33.3)		1 (33.3)		3 (50)	1 (16.7)	5 (41.7)	1 (8.3)
Fatigue			2 (66.7)		3 (50)	1 (16.7)	5 (41.7)	1 (8.3)
Hypokalemia			1 (33.3)		3 (50)		4 (33.3)	
Hypothyroidism	1 (33.3)		1 (33.3)		3 (50)		5 (41.7)	
Vomiting					3 (50)		3 (25)	
ALT increased					2 (33.3)	1 (16.7)	2 (16.7)	1 (8.3)
AST increased	1 (33.3)				2 (33.3)	1 (16.7)	3 (25)	1 (8.3)
Hoarseness					2 (33.3)		2 (16.7)	
Hypertension	1 (33.3)	1 (33.3)			2 (33.3)	2 (33.3)	3 (25)	3 (25)
Weight loss	1 (33.3)		1 (33.3)		2 (33.3)		4 (33.3)	
Abdominal pain	1 (33.3)				1 (16.7)		2 (16.7)	
Dry mouth	1 (33.3)				1 (16.7)		2 (16.7)	
Dry skin	1 (33.3)				1 (16.7)		2 (16.7)	
Epistaxis					1 (16.7)		1 (8.3)	
Upset stomach					1 (16.7)		1 (8.3)	
Cold Sores					1 (16.7)		1 (8.3)	
Headache					1 (16.7)		1 (8.3)	
Hyponatremia					1 (16.7)	1 (16.7)	1 (8.3)	1 (8.3)
Insomnia					1 (16.7)		1 (8.3)	
Nail discoloration					1 (16.7)		1 (8.3)	
Nausea			1 (33.3)		1 (16.7)		2 (16.7)	
Oral dysesthesia	1 (33.3)				1 (16.7)		2 (16.7)	
Pruritus					1 (16.7)		1 (8.3)	
Rash maculo-papular	1 (33.3)		3 (100)	1 (33.3)	1 (16.7)		5 (41.7)	1 (8.3)
Skin irritation					1 (16.7)		1 (8.3)	
Skin wound					1 (16.7)		1 (8.3)	
Skin hyperpigmentation					1 (16.7)		1 (8.3)	
Skin ulceration					1 (16.7)		1 (8.3)	
Anemia			1 (33.3)	1 (33.3)			1 (8.3)	1 (8.3)
Arthralgia	1 (33.3)						1 (8.3)	
Bruising			1 (33.3)				1 (8.3)	
Cough			1 (33.3)				1 (8.3)	
Dizziness			1 (33.3)				1 (8.3)	
Gastroesophageal reflux disease			1 (33.3)				1 (8.3)	
Muscle cramping	1 (33.3)						1 (8.3)	
Hand-foot sensitivity			1 (33.3)				1 (8.3)	
Face rash			1 (33.3)				1 (8.3)	
Skin hypopigmentation	1 (33.3)						1 (8.3)	
Stomach pain	1 (33.3)						1 (8.3)	

€ = there were no grade 5 events in any patient.

(A) 20 mg cabozantinib cohort (*n* = 3): Most common AE of any grade attributed to cabozantinib of 20 mg/day was diarrhea (*n* = 3/3, 100%). The most common AE of grades 3-5 attributed to cabozantinib of 20 mg/day were hypertension, and thromboembolic event with one event each (*n* = 1/3, 25%).

(B) 40 mg cabozantinib cohort (*n* = 3): Most common AE of any grade attributed to cabozantinib of 40 mg/day was maculopapular rash (*n* = 3/3, 100%). AEs of grades 3-5 attributed to cabozantinib of 40 mg/day were thromboembolic events (*n* = 2/3, 66.7%), anemia (*n* = 1/3, 33.3%), diarrhea (*n* = 1/3, 33.3%), and maculopapular rash (*n* = 1/3, 33.3%).

(C) 60 mg cabozantinib cohort (*n* = 6): The 3 most common AEs of any grade attributed to cabozantinib of 60 mg/day were diarrhea (*n* = 6/6, 100%), palmar-plantar erythrodysesthesia syndrome (*n* = 5/6, 83.3%), thromboembolic events (*n* = 5/6, 83.3%), dysgeusia (*n* = 4/6, 66.7%), and oral mucositis (*n* = 4/6, 66.7%). The 2 most common AEs of grades 3-5 attributed to cabozantinib of 60 mg/day were hypertension (2/6, 33.3%), and thromboembolic events (2/6, 33.3%).

##### AEs Attributed to Avelumab

The immune-related adverse events (irAEs) attributed to avelumab are described in Table 4. Combining all the 3 cohorts, diarrhea (*n* = 4/12, 33.3%), fatigue (*n* = 4/12, 33.3%), maculopapular rash (*n* = 3/12, 25%), and palmar-plantar erythrodysesthesia syndrome (*n* = 3/12, 25%) were the most common irAEs of any grade noted during the study period. Similarly, combining all the 3 cohorts, 5 patients were noted to have grade 3 or more irAEs which were diarrhea (*n* = 2/12, 16.7%), acute kidney injury (*n* = 1/12, 8.3%), fatigue (*n* = 1/12, 8.3%), hypercalcemia (*n* = 1/12, 8.3%), hypocalcemia (*n* = 1/12, 8.3%), and maculopapular rash (*n* = 1/12, 8.3%).

**Table 4.** Incidence of treatment-related adverse events attributed to avelumab.

	Cabozantinib 20 mg plus avelumab (*n* = 3)		Cabozantinib 40 mg plus avelumab (*n* = 3)		Cabozantinib 60 mg plus avelumab (*n* = 6)		Total (n-=12)	
Adverse events, *n* (%)	Any grade	Grades 3-5	Any grade	Grades 3-5	Any grade	Grades 3-5	Any grade	Grades^€^ 3-5
Diarrhea			1 (33.3)		3 (50)	2 (33.3)	4 (33.3)	2 (16.7)
Acute kidney injury					2 (33.3)	1 (16.7)	2 (16.7)	1 (8.3)
Fatigue			2 (66.7)		2 (33.3)	1 (16.7)	4 (33.3)	1 (8.3)
Allergic rhinitis					1 (16.7)		1 (8.3)	
Colitis					1 (16.7)		1 (8.3)	
Hypercalcemia					1 (16.7)	1 (16.7)	1 (8.3)	1 (8.3)
Hypocalcemia					1 (16.7)	1 (16.7)	1 (8.3)	1 (8.3)
Palmar-plantar erythrodysesthesia syndrome	1 (33.3)		1 (33.3)		1 (16.7)		3 (25)	
Pruritus	1 (33.3)				1 (16.7)		2 (16.7)	
Skin hyperpigmentation					1 (16.7)		1 (8.3)	
Anorexia			1 (33.3)				1 (8.3)	
Cough			1 (33.3)				1 (8.3)	
Dry skin	1 (33.3)						1 (8.3)	
Dysgeusia			1 (33.3)				1 (8.3)	
Hypothyroidism	1 (33.3)		1 (33.3)				2 (16.7)	
Plantar Fascitis	1 (33.3)						1 (8.3)	
Rash maculopapular			3 (100)	1 (33.3)			3 (25)	1 (8.3)
Face lesions			1 (33.3)				1 (8.3)	
Skin hypopigmentation	1 (33.3)						1 (8.3)	

€ = there were no grade 5 events in any patient.

Table 5 describes all the SAEs encountered by the patients from various cohorts. No patient developed SAE in 20 mg cabozantinib cohort. One patient had 3 SAEs in 40 mg cohort: fall, hypoglycemia, and pulmonary embolism (PE), respectively, with PE being related to cabozantinib. Five patients had SAEs in the 60 mg cohort, including acute kidney injury, hypercalcemia, and hypocalcemia were related to avelumab. One patient with epistaxis and 4 patients with five thromboembolic events (2 PE, 2 DVT, one nonocclusive thrombus within the inferior vena cava) in 60 mg cohort were attributed to cabozantinib. No dose-limiting toxicity (DLT) was observed in any of the 12 patients.

**Table 5.** Incidence of serious adverse events (SAE).

CTCAE term, number of patients (%)	All SAEs Cabozantinib 20 mg plus avelumab (*n* = 3)	All SAEs Cabozantinib 40 mg plus avelumab (*n* = 3)	All SAEs Cabozantinib 60 mg plus avelumab (*n* = 6)	All SAEs (%) (*n* = 12)
Acute kidney injury ^†^			1 (16.7)	1 (8.3)
Cardiac conduction disorder			1 (16.7)	1 (8.3)
Epistaxis^*^			1 (16.7)	1 (8.3)
Fall		1 (33.3)		1 (8.3)
Hypercalcemia ^†^			1 (16.7)	1 (8.3)
Hypocalcemia^†^			1 (16.7)	1 (8.3)
Hypoglycemia		1 (33.3)		1 (8.3)
COVID-19 infection			1 (16.7)	1 (8.3)
Lung Infection			1 (16.7)	1 (8.3)
Sepsis			1 (16.7)	1 (8.3)
Cellulitis			1 (16.7)	1 (8.3)
Thromboembolic event^*,µ^		2 (66.6)	5 (83.3)	7 (58.3)
Total patients affected	0	1 (33.3)	5 (83.3)	6 (50)

^*^Related to cabozantinib; ^†^related to avelumab; ^µ^6 of the 7 thromboembolic SAEs were related to cabozantinib (4 = possible, one = probable, one = definite).

### Drug Interruptions

Drug interruptions occurred in 9 patients (*n* = 9/12, 75%), including 3 in 40 mg cabozantinib cohort and 6 in 60 mg cabozantinib cohort. Six patients (*n* = 6/12, 50%) required dose reductions of cabozantinib after the completion of the DLT period: one in the 40 mg cohort and 5 in the 60 mg cohort. Dose reductions in cabozantinib were due to oral mucositis (*n* = 1) and hand-foot syndrome (*n* = 4). Avelumab was held and tretment with corticosteroids was required in 3 patients due to immune-related colitis (*n* = 2) and nephritis (*n* = 1). No patients permanently discontinued cabozantinib due to toxicity. One patient permanently discontinued avelumab due to grade 3 nephritis. One patient died due to sudden cardiac arrest, which was clinically suspected to be caused by pulmonary embolism related to disease, and not due to the trial drugs during the study period. This patient had a level III tumor thrombus at baseline.

**Table:** 

Secondary Assessment Method	
Title	Efficacy and survival
Number of patients screened	19
Number of patients enrolled	12
Number of patients evaluable for toxicity	12
Number of patients evaluated for efficacy	12
Evaluation method	RECIST 1.1
Response assessment, CR	1 (8.3%)
Response assessment, PR	5 (41.6%)
Response assessment, SD	5 (41.6%)
Response assessment, PD	1 (1%)
Median duration assessment, PFS	11.3 months (CI: 7.7-33.1 months)
Median duration assessment, response duration	20.6 months (13.9-31.2 months)
Median duration assessment, duration of treatment	19 months (4.4-31.2 months)
Outcome notes, efficacy	The median follow-up time was 27.6 months (range 4.4-43.5 months), and the median duration of treatment was 19 months (range 4.4-31.2 months). The ORR was 50%, with one complete response (CR) and 5 partial responses (PR). The disease control rate was 92% (*n* = 11/12) of the patients (Fig. 2). One patient had PD due to new osseous metastasis, though target lesions decreased in size at the same time. Radiological PFS at 6 and 12 months was reported in 67.7% and 33.5% of patients, respectively. Treatment was continued in 5 patients beyond progression (41.7%) (Fig. 3). The median PFS was 11.3 months (95% CI: 7.7-33.1 months) (Fig. 4 ).

**Figure 2.**
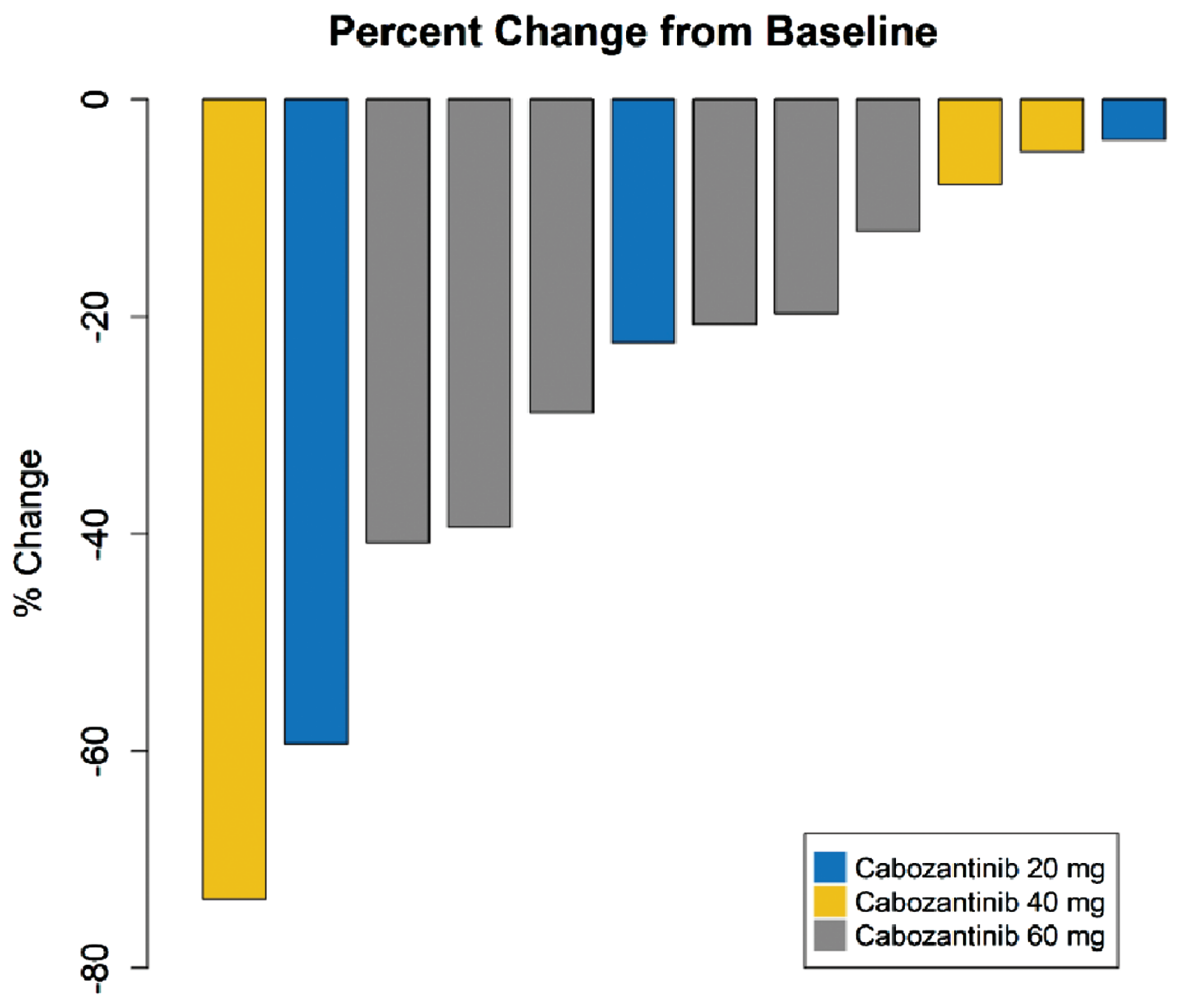
Plot of confirmed tumor regression from baseline as measured by RECIST in all evaluable patients (Waterfall plot of disease response).

**Figure 3.**
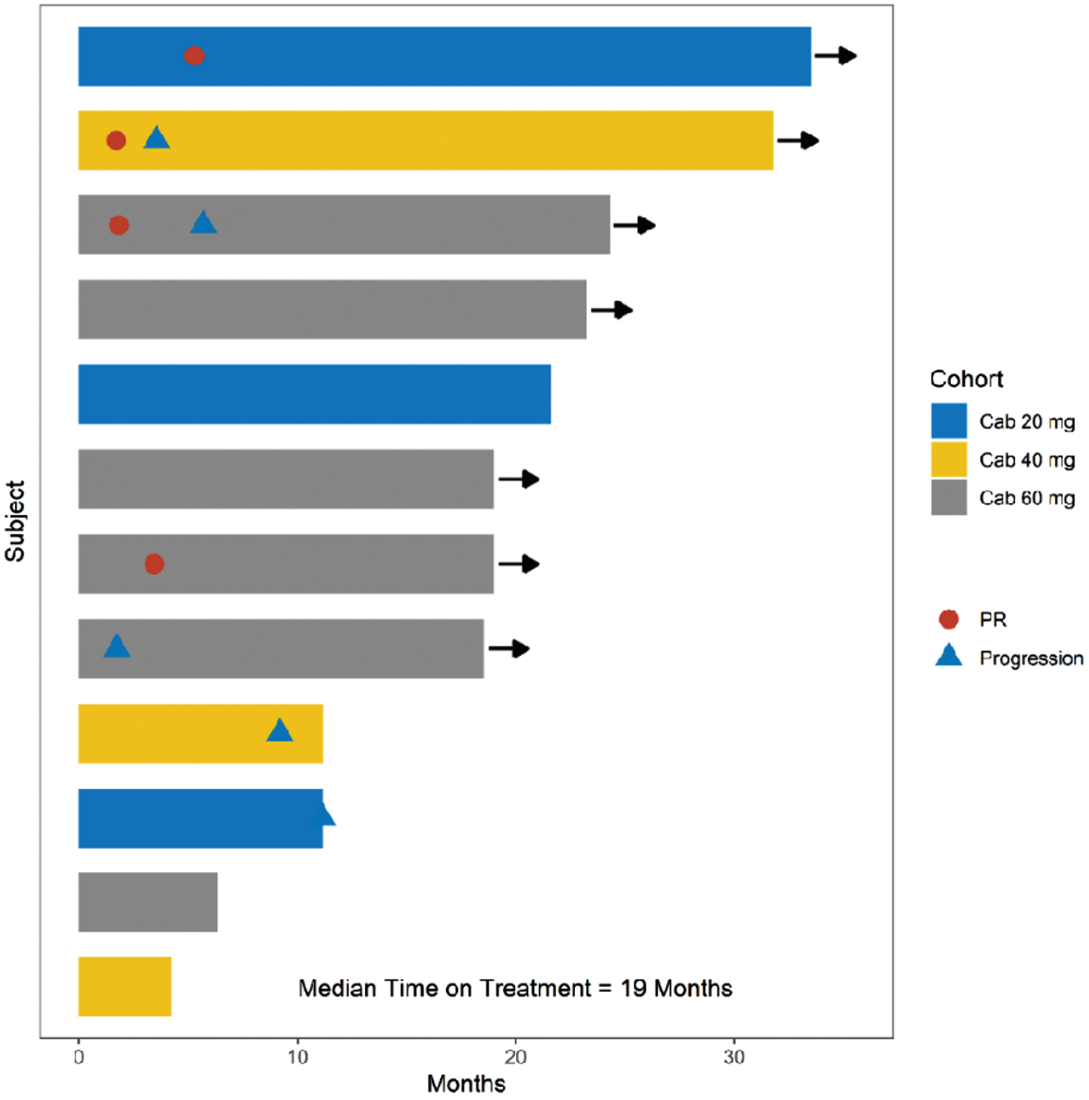
Duration of treatment with avelumab and cabozantinib in all 3 cohorts with objective responses (Swimmer plot of disease response and treatment duration).

**Figure 4.**
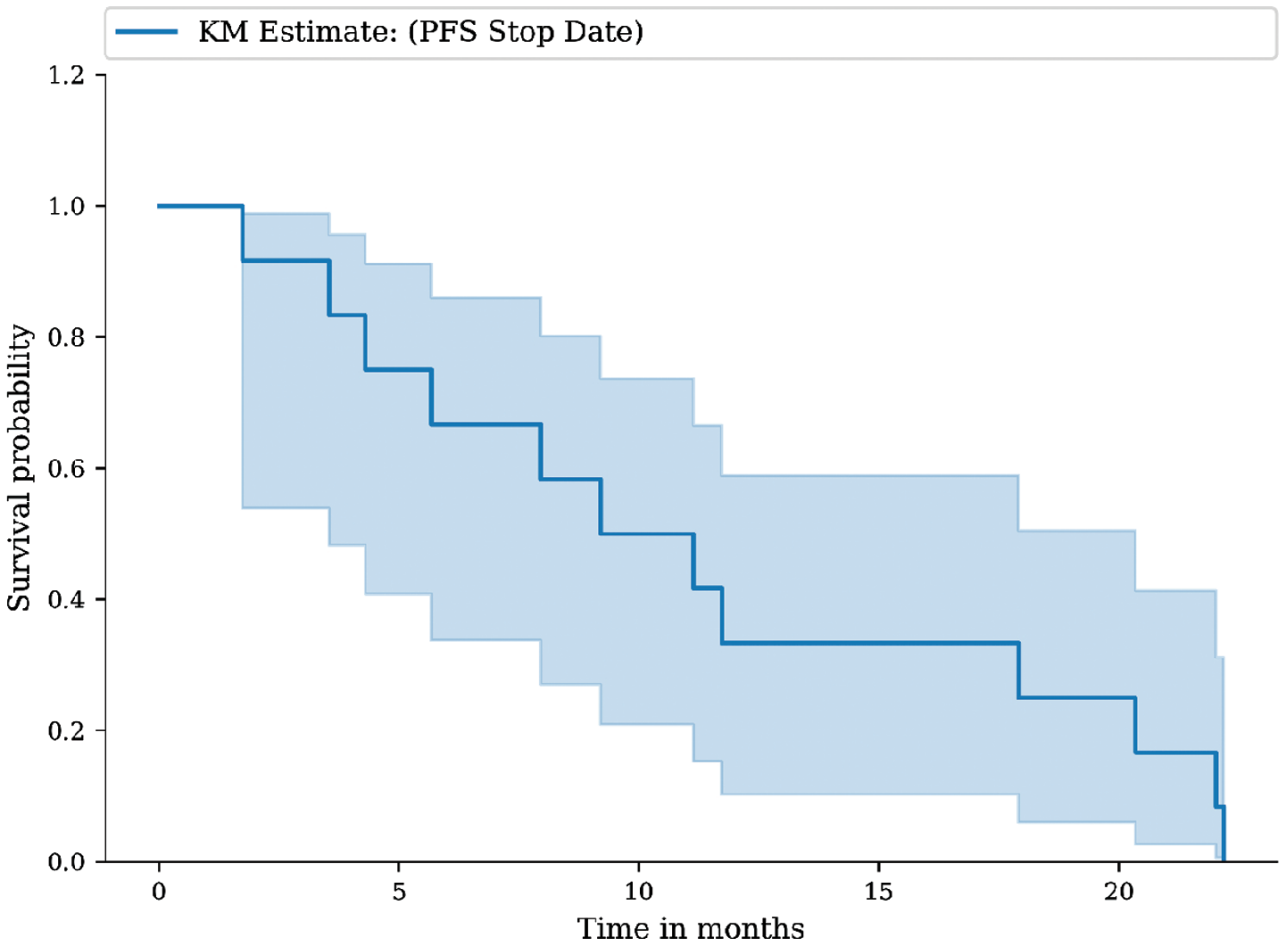
Kaplan-Meier estimates of progression-free survival (PFS) for the overall study population.

## Assessment, Analysis, and Discussion

**Table:** 

Completion	Study completed
Investigator’s assessment	Active and should be pursued further

This phase I study demonstrates that the combination of avelumab and cabozantinib is safe with manageable toxicities in patients with mccRCC. No DLT was observed in any of the patients enrolled in this phase I study. However, 6 patients (*n* = 6/12, 50%) did require dose reductions of cabozantinib after the DLT window, including 5 of 6 patients treated with cabozantinib 60 mg/day. Due to a high incidence of persistent grade 2 toxicities at cabozantinib 60 mg/day, the RP2D of cabozantinib is 40 mg/day. The adverse-event profile of avelumab and cabozantinib was consistent with the previous studies of each agent as monotherapy, and no new safety concerns were identified.^3,11,12^ The rate of grade 3 treatment-related adverse events (TRAEs) was acceptable, and no grade ≥ 4 TRAEs were reported. No DLTs were reported, and an MTD was also not reached during the DLT window period.

The venous thromboembolism (VTE) rate in our study was notably high. Due to the single-arm design of the study, it is impossible to accurately determine the relative contribution from the disease, cabozantinib, and/or avelumab. Metastatic RCC is known to cause a hypercoagulable state predisposing to VTE.^13^ In this study, we included patients with baseline tumor thrombus, a known risk factor for VTE, and patients with a history of VTEs. Additionally, cabozantinib has been reported to increase the incidence of VTEs in prior studies.^3,6,14^ In the CheckMate 9ER trial, the incidence of VTEs with cabozantinib plus nivolumab combination was 7% (including 2% PE).^6^ Avelumab is only rarely associated with VTEs.^15,16^ However, it is not possible to entirely rule out the potential for synergistic toxicity when added to cabozantinib due to the single-arm design of this trial. Most likely, the high VTE rate in our study is related to the underlying diagnosis of mccRCC, with inherent biologic characteristics (eg, presence of tumor thrombus, a history of VTEs in our cohort), and an added contribution from cabozantinib.

The combination demonstrated preliminary anti-tumor activity in our study. After a median period of 19 months of treatment, the reported ORR was 50% (*n* = 1 CR, and *n* = 5 PR), and DCR was 92% (*n* = 11/12) which is approximately similar to the Checkmate 9 ER study that showed ORR of 55.7% and DCR of 68%.^6^ It is notable that 5 patients in our study were treated beyond progression, suggesting that clinical benefit after progression or pseudo-progression can be observed in some patients with this combination. Previous studies conducted on cabozantinib in combination with other PD-1/PD-L1 therapies also concluded the RP2D of cabozantinib to be 40 mg/day.^17,18^ Currently, 3 combination regimens of tyrosine kinase inhibitor/immunotherapy are approved for the first-line treatment in mccRCC.^4-6^ In the CheckMate 9ER phase III trial [nivolumab plus cabozantinib (40 mg/day)], at a median follow-up of 18.1 months, the median PFS was 16.6 months with an ORR of 55.7%.^6^ In the KEYNOTE-426 phase III trial [pembrolizumab and axitinib], at a median follow-up of 18.1 months, the median PFS was 15.1 months with an ORR of 59.3%.^5^ Similarly, in JAVELIN Renal 101, avelumab plus axitinib combination showed a median PFS of 13.8 months, and ORR of 55.2%. These findings are similar to the secondary endpoints of our study, with a median PFS of 11.3 months and ORR of 50% after a median follow-up of 27.6 months, highlighting the likely efficacy of this combination.

Overall, the combination of avelumab and cabozantinib showed an acceptable safety profile, an acceptable rate of grade 3/4 TRAEs, and a preliminary anti-tumor activity against mccRCC. However, this combination is unlikely to be developed further in the mccRCC setting because of multiple other combinatorial regimens of cabozantinib plus immune checkpoint inhibitors either garnering regulatory approval (CheckMate 9ER trial), or reaching advanced phases of development in the registration trials (NCT03937219, NCT04338269).^19,20^ Having said that, there is an ongoing NCI-funded phase III Alliance trial (MAIN-CAV trial) utilizing this combination of cabozantinib plus avelumab as the maintenance regimen in the metastatic urothelial carcinoma setting.^10^

In this phase I study involving patients with previously untreated advanced mccRCC, avelumab, and cabozantinib was found to be safe and demonstrated tolerable AEs with preliminary evidence of anti-tumor activity. Even though the DLT was not met in any of the 3 cohorts, the recommended RP2D dose for cabozantinib is 40 mg/day due to a high incidence of intolerable grade 2 toxicity for cabozantinib 60 mg/day.

## Data Availability

The data underlying this article will be shared on reasonable request to the corresponding author.
